# A case-series study to explore the efficacy of foot orthoses in treating first metatarsophalangeal joint pain

**DOI:** 10.1186/1757-1146-3-17

**Published:** 2010-08-27

**Authors:** Brian J Welsh, Anthony C Redmond, Nachiappan Chockalingam, Anne-Maree Keenan

**Affiliations:** 1 Musculoskeletal and Rehabilitation Services, NHS Leeds Community Healthcare, St Mary's Hospital, Leeds, LS12 3QE, UK; 2 NIHR Leeds Musculoskeletal Biomedical Research Unit and Section of Musculoskeletal Disease, University of Leeds, 2nd Floor, Chapel Allerton Hospital, Leeds LS7 4SA, UK; 3 Faculty of Health, Staffordshire University, Stoke on Trent ST4 2DF, UK

## Abstract

**Background:**

First metatarsophalangeal (MTP) joint pain is a common foot complaint which is often considered to be a consequence of altered mechanics. Foot orthoses are often prescribed to reduce 1^st ^MTP joint pain with the aim of altering dorsiflexion at propulsion. This study explores changes in 1^st ^MTP joint pain and kinematics following the use of foot orthoses.

**Methods:**

The effect of modified, pre-fabricated foot orthoses (X-line^®^) were evaluated in thirty-two patients with 1^st ^MTP joint pain of mechanical origin. The primary outcome was pain measured at baseline and 24 weeks using the pain subscale of the foot function index (FFI). In a small sub-group of patients (n = 9), the relationship between pain and kinematic variables was explored with and without their orthoses, using an electromagnetic motion tracking (EMT) system.

**Results:**

A significant reduction in pain was observed between baseline (median = 48 mm) and the 24 week endpoint (median = 14.50 mm, z = -4.88, p < 0.001). In the sub-group analysis, we found no relationship between pain reduction and 1^st ^MTP joint motion, and no significant differences were found between the 1^st ^MTP joint maximum dorsiflexion or ankle/subtalar complex maximum eversion, with and without the orthoses.

**Conclusions:**

This observational study demonstrated a significant decrease in 1^st ^MTP joint pain associated with the use of foot orthoses. Change in pain was not shown to be associated with 1^st ^MTP joint dorsiflexion nor with altered ankle/subtalar complex eversion. Further research into the effect of foot orthoses on foot function is indicated.

## Background

First metatarsophalangeal (MTP) joint pain is a common foot complaint, often associated with osteoarthritis (OA): with more than 20% of people over the age of 40 reporting 1^st ^MTP joint OA pain [1]. Altered kinematics at the foot and ankle have been suggested to be associated with such 1^st ^MTP joint pain which, in turn, may act as a precursor to osteoarthritic changes at the 1^st ^MTP joint [2,3]. Authors have proposed that failure of the first metatarsal to achieve sufficient plantarflexion, prior to the propulsive phase of the gait cycle, may prevent the posterior glide of the metatarsal head along its sesamoid apparatus [4,5]. This is thought to result in abnormal hallux dorsiflexion, which terminates with impingement between the dorsal articular surfaces of the 1^st ^metatarsal and the proximal phalanx, with resulting pain and inflammation within the joint capsule [6,7].

It is also suggested that this functional loss of hallux dorsiflexion at the 1^st ^MTP joint can occur despite the fact that adequate dorsiflexion is available when the joint is assessed in a non-weightbearing condition. This functional, as opposed to structural, blockade has been termed functional hallux limitus (FHL). Laird [8] defined this concept as non-weightbearing dorsiflexion greater than 50° at the 1^st ^MTP joint, with less than 14° 1^st ^MTP joint dorsiflexion at terminal stance. Others have given descriptions, investigated FHL and offered their own interpretation of the condition [9-14].

While 1^st ^MTP joint pain is often associated with OA, this is not the only cause of pain. The term "mechanical joint pain" is used commonly within the rheumatology literature and refers to pain that is mechanical in origin or influence when there is a pattern of increased symptoms with weightbearing activities and when other potential systematic causes have been excluded [15]. More recently, this term has been extended to include foot pain and given the importance placed on 1^st ^MTP joint function, it readily extends itself to 1^st ^MTP joint pain.

Foot orthoses are thought to decrease mechanically induced 1^st ^MTP joint pain by allowing the 1^st ^metatarsal to achieve sufficient plantarflexion in preparation for propulsion [16]. Whilst the relationship between ankle/subtalar joint pronation and 1^st ^MTP joint pain is unclear, clinicians commonly prescribe foot orthoses with medial posting to alter the degree and timing of ankle/subtalar complex pronation in the treatment of 1^st ^MTP joint pain. First ray cut outs and forefoot postings are further orthotic modifications that have been employed to improve 1^st ^ray function and reduce pain.

There are, however, limitations in the evidence to support such approaches. Despite sound reasoning and theoretical principles, the approach is largely based on subjective justification [16] or single case design [4,17,18]. The existing literature has also focused primarily on normal or asymptomatic participants [19-25]. Furthermore, the relationship between pain and foot function has not been explored.

The aim of this study was to investigate change in 1^st ^MTP joint pain levels when foot orthoses are prescribed with the rationale of increasing 1^st ^MTP joint dorsiflexion. Relationships between changes in 1^st ^MTP joint pain levels and changes in the mechanical effects of foot orthoses on 1^st ^MTP joint and ankle/subtalar complex kinematics were also explored in a small number of participants.

## Methods

This two part study was undertaken at St James' and Chapel Allerton Hospitals, Leeds, United Kingdom. Ethical approval was granted from the Faculty of Health and Sciences Independent Peer Review Panel, Staffordshire University and Leeds West Local Research Ethics Committee. The two parts of this study were (i) an investigation of the clinical effects of foot orthoses on mechanical joint pain at the 1^st ^MTP joint; and (ii) an exploration of the mechanical effects of the foot orthoses in a small sub-group of the same participants.

### Participants

Thirty five participants (mean age, 42 years; range 21-63 years) were recruited from primary care referrals received within Musculoskeletal and Rehabilitation Services and the Community Podiatry Service, Leeds, United Kingdom. Participants were recruited who had mechanically induced 1^st ^MTP joint pain which was required to be of at least 4 weeks duration and at a level of at least 40 mm on a 100 mm visual analogue pain scale (VAPS) as previously described [26], which was considered an appropriate pain level to warrant intervention [27].

As the foot orthoses being tested in the study were to be modified to offer a tailored level of pronatory control, participants were required to demonstrate a Foot Posture Index (FPI-6) score of greater than 4/12 [28].

Participants were excluded if they had established hallux valgus, a previous history of foot and ankle trauma, fracture or surgery, or an existing diagnosis of inflammatory, metabolic, neurological or vascular disease. Individuals who exhibited less than 40° of available 1^st ^MTP joint dorsiflexion, measured by a non-weightbearing technique previously described by Buell et al [29], were also excluded from the study as this has been reported to be the range of 1^st ^MTP joint dorsiflexion used during normal propulsion [10,21,22] and would therefore have indicated structural limitation at the joint. Where participants reported bilateral 1^st ^MTP joint pain, the joint which gave the most pain was selected for the purposes of the study.

The sample size was based on Pitman's Asymptotic Relative Efficiency [30]. A calculation was performed using a standard deviation of 17.8, based on previous work that used the same outcome measure as this study [31]. A sample size of 32 participants provided 80% power to detect a 10 mm difference at an alpha level of 0.05. This was increased to 35 to allow for approximately 10% drop-out [32]. A reduction of 10 mm on the pain subscale of the FFI was chosen *a priori *as a clinically relevant change, based on previous data [33,34].

### Foot orthoses

**Figure 1:**
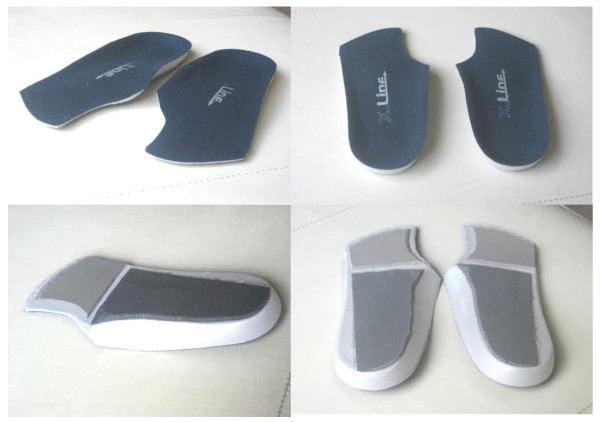
**Type of foot orthoses used in the study**. This shows the pre-fabricated foot orthoses that were used in the study, following individually tailored modification.

All patients were prescribed pre-fabricated, foot orthoses (X-line^®^, Healthystep, Mossley, UK). Sagittal and frontal plane pronatory control was increased using high density (400 kg/m^3^) ethyl-vinyl acetate wedged posting, adhered to the medial underside of the foot orthoses. The posting was tailored to each individual's requirements as determined by a standard clinical evaluation by an experienced musculoskeletal specialist podiatrist (BJW). Adequacy of pronatory control provided by the foot orthoses was assessed through a reduction in FPI-6 score of at least 2 points. All foot orthoses were cut to the level of the toe sulci. The first metatarsal head region of the foot orthoses was cut out and a forefoot extension of 3 mm open cell polyurethane foam was added as in Figure 1. It is acknowledged that a pragmatic prescription protocol increases variability in device prescription, but the tailoring of prescriptions to the patient's specific needs reflects a more realistic therapeutic approach than the use of artificially standardised prescriptions.

### Clinical Outcome Measures

The primary outcome for this study was pain measured using a modification of the pain subscale of the Foot Function Index (FFI) [35], with an endpoint of 24 weeks. This instrument has demonstrated good test-retest reliability, internal consistency and both construct and criterion validity [35]. While the FFI was developed to assess the effectiveness of foot orthoses on foot pathology in people with rheumatoid arthritis (RA), it has been widely used to investigate non-RA populations [31,36-38] and was deemed suitable for this study.

Secondary outcomes were 1^st ^MTP joint and ankle/subtalar complex motions derived from an electromagnetic motion tracking (EMT) system, as described previously by Halstead et al [22] and Longworth et al [39].

### Clinical protocol

At initial contact, a clinical assessment was conducted and baseline outcomes were captured. Participants were provided with guidance on how to complete the FFI pain subscale, in relation to their 1^st ^MTP joint pain, prior to data capture. The pre-fabricated foot orthoses used in the study were modified as described above and fitted into the participant's shoes. Foot orthoses were issued only if footwear was appropriate, determined by an assessment tool devised by Menz and Sherrington [40]. In addition to the footwear assessment tool, heel counter height was also assessed which was considered an important factor in the provision of foot orthoses. Verbal and written advice was issued to each participant, detailing important information about the wearing of foot orthoses.

While the primary endpoint was 24 weeks, participants underwent interim clinical review after 8 weeks, at which time they were asked to complete again the pain subscale of the FFI. Further postal FFI pain subscale questionnaires were administered, and telephone reviews were conducted, at 12 weeks as well as at the 24 week endpoint.

### Gait Analysis

At the 8 week review, 10 participants were invited to participate in the 2^nd ^stage of the study exploring the relationship between pain and kinematic outcomes. Individuals were invited on the basis of the extent of any benefit that had been gained from the wearing of the foot orthoses at this stage of the trial. A sample was constructed to include a range of participants, from those who had gained much pain relief, to those who had gained the least benefit. The intention was to explore the relationship between change in pain and kinematic response for the indexed (or painful) foot.

Joint kinematic data for the 1^st ^MTP joint and the ankle/subtalar complex was collected using a Fastrak™ EMT system with a long-range transmitter (Polhemus Inc., Colchester, VT). The long-range configuration produces a low frequency electromagnetic field, with a radius of approximately two metres from the transmitter, which was centred along a walkway. The four metre walkway was raised from the floor of the gait laboratory to prevent metallic interference. Four sensors were used, capturing at 30 Hz. Sensors were attached to the hallux and 1^st ^metatarsal to derive sagittal plane motion data for the 1^st ^MTP joint. Sensors were also attached to the posterior calcaneus and medial tibia to derive ankle/subtalar complex motion data. The 1^st ^metatarsal sensor was attached using a Velcro strap in accordance with a protocol devised by Longworth et al [39]. This reduces potential error due to extensor hallucis longus contraction during hallux dorsiflexion, as previously described by Umberger et al [41]. The other sensors were attached with double-sided tape, and secured with Hypafix™ tape over the sensors, to anatomical sites with minimal overlying soft tissue to reduce possible sensor movement during walking. All cables were secured with straps to the limb and waist with a belt. The 6 D Research™ software package (Skill Technologies, Phoenix, AZ, USA) was used to post-process the data from the EMT sensors as described previously [42].

Angular rotation between the anatomically mounted sensors was determined using a previously described joint co-ordinate system [10,42]. The joint motions investigated were: x-axis maximum stance phase dorsiflexion at the 1^st ^MTP joint and y-axis maximum stance phase eversion at the ankle/subtalar complex. 6DNorm software (M.Cornwall, Northern Arizona University, Flagstaff, AZ, USA) was used to generate motion-time curves, normalized to 100% of the gait cycle, for each axis of rotation.

**Figure 2:**
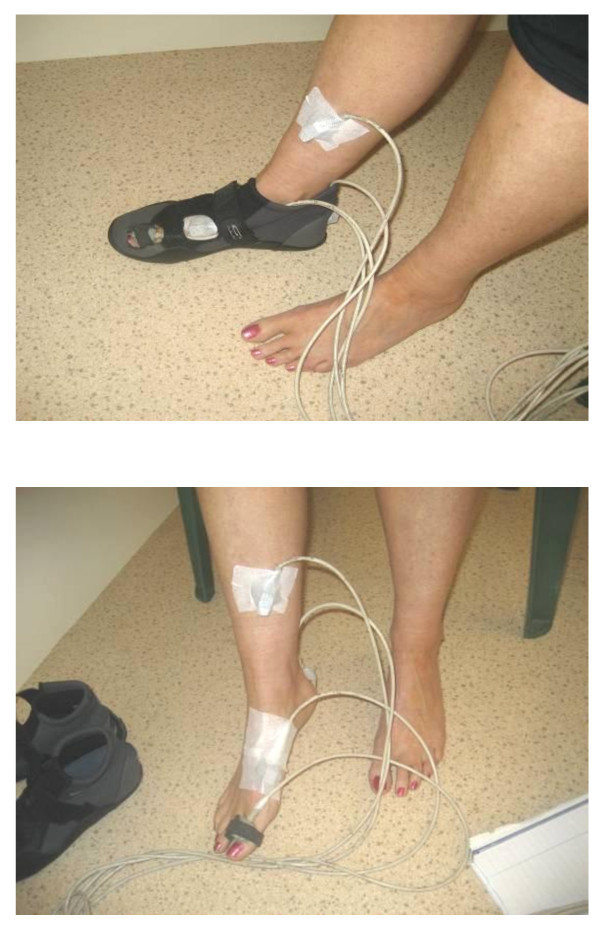
**2a. Sensor placement. 2b. With neoprene boot**. This shows the position of the EMT sensors attached at the anatomical landmarks and with the Velcro fastening, neoprene boot secured, which was used during the capture of kinematic data.

**Figure 3:**
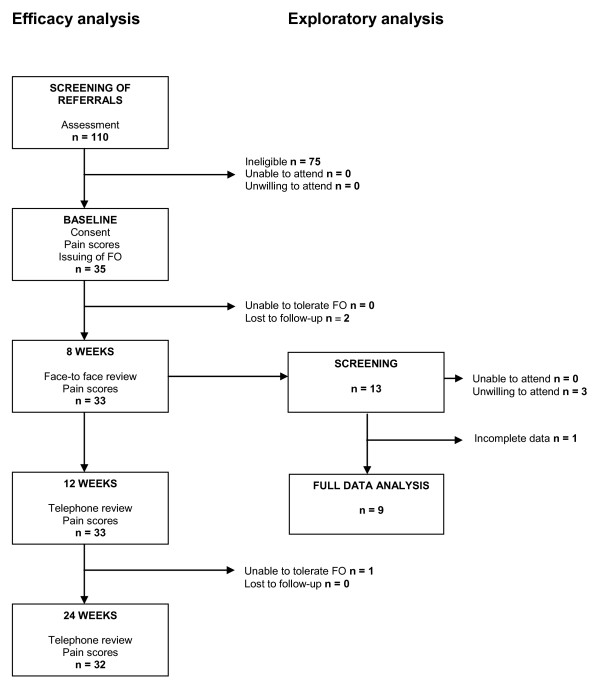
**Participant flow diagram**. This shows the journey for all participants through the study protocol including both efficacy analysis, which all participants took part in, and the exploratory analysis, which a selected cohort took part in.

EMT sensors were secured as indicated in Figure 2a. Participants wore Velcro fastening, neoprene boot, of appropriate size, similar to a previously used protocol [31]. The flexibility of the boots minimised the confounding effects of structured footwear and allowed windows to be cut into the footwear so that the hallux, 1^st ^metatarsal and calcaneal placed sensors were not disturbed or displaced during data capture (figure 3b). For calibration or 'boresighting' participants stood near the centre of the electromagnetic field, with the calcaneus vertical and talar head palpable equally on both medial and lateral sides to establish a reference position [43]. This 'boresighting' procedure has been described previously [42].

When comfortable with the set-up, participants initiated gait for one metre at a self-selected speed, before entering the 1.5 metre calibrated capture volume and continued walking for a further 1.5 metres once through the volume. Three trials were completed for each of the experimental conditions 'orthoses' and 'no-orthoses'. Care was taken when inserting the foot orthoses into, and removing them from, the boots, so that the sensors were not disturbed or displaced. This was enabled by the Velcro fastening. The order of data collection was randomized. Data from the three trials of the indexed limb was normalised to percentiles of the gait cycle and averaged to maximise within-subject consistency. The original dataset of 10 was reduced to 9 due to technical problems.

### Data analysis

All analyses were conducted using Microsoft Excel™ and SPSS version 15 for Windows. The analysis strategy was divided into two phases, an efficacy phase and an exploratory phase. For the efficacy analysis, comparisons of pain scores between baseline and primary endpoint (24 weeks) were explored descriptively and using a Wilcoxon's Signed Rank test. The exploratory analysis investigated a subset of patients (n = 9) who attended the gait laboratory for detailed kinematic studies. For the exploratory analysis, data were explored descriptively and using Wilcoxon's Signed Rank test. Relationships between variables were explored graphically and using Spearman's Rho. Additionally, a difference in response between 1^st ^MTP joint dorsiflexion and ankle/subtalar complex maximum eversion was explored using descriptive statistics and then using a Mann-Whitney U test. P values < 0.05 were considered significant.

## Results

Table 1 shows the participant characteristics at baseline. Complete data were obtained from 32 participants in the efficacy phase (6 male:26 female) and from nine in the exploratory phase, as described in the participant flow diagram, Figure 3. Data was obtained for the left index limb for 14 participants and for the right index limb in 18 participants. At baseline, there were no significant correlations between reported pain and the following: age (r = -0.231, p = 0.203), body mass index (r = -0.155, p = 0.397), 1^st ^MTP joint dorsiflexion (r = 0.24, p = 0.895) or FPI-6 (r = 0.273, p = 0.130).

**Table 1:** Participant characteristics at baseline

N = 35	Mean	Std Dev.
Age (years)	42.2	± 11.5
Body mass index	24.4	± 3.8
Duration of symptoms (months)	26.3	± 30.8
1^st ^metatarsophalangeal joint range of motion: non-weightbearing	63.5°	± 15.2°
Foot Posture Index-6 left: baseline	7.3	± 2.0
Foot Posture Index-6 right: baseline	7.0	± 2.2

### Efficacy analysis

**Figure 4:**
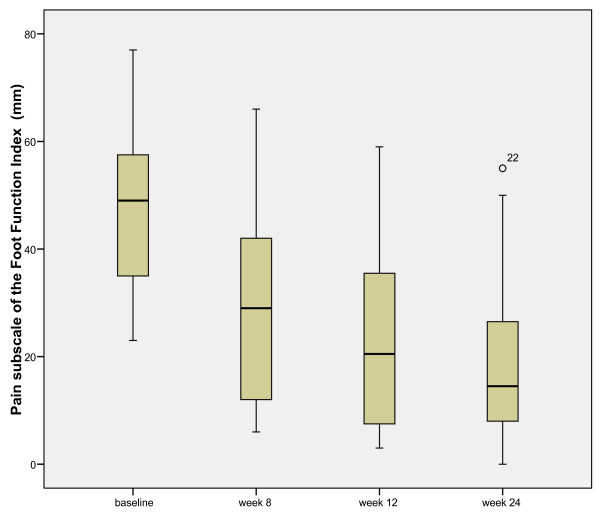
**Distribution of the reduction in FFI (pain) scores from baseline to week 24 (0 = no pain and 100 = worst pain imaginable)**. This shows the systematic reduction in pain scores over the treatment period.

Following the introduction of the treatment foot orthoses, there was a significant reduction in median pain score from baseline (48 mm) to 24 weeks (14.5 mm, z = -4.88, p < 0.001). Figure 4 illustrates the systematic reduction in pain scores at baseline, eight weeks (29 mm), 12 weeks (20.5 mm) and the 24 week endpoint.

### Exploratory analysis

The exploratory kinematic analysis indicated that the pain reduction reported by participants between pre-intervention baseline scores and post-intervention scores was not accompanied by any systematic change in 1^st ^MTP joint dorsiflexion or ankle/subtalar complex pronation (eversion). After wearing foot orthoses for 8 weeks, there was no systematic change in maximum 1^st ^MTP joint dorsiflexion during the walking cycle (no-orthoses median = 8°, IQR = 22.1 vs orthoses median = 7°, IQR = 23.3, p = 0.954). Similarly, there was no consistent effect of the foot orthoses on maximum ankle/subtalar complex eversion (no-orthoses median = 1°, IQR = 8.9 vs orthoses median = 1°, IQR = 6.4, p = 0.672).

## Discussion

The aim of this study was to explore the efficacy of orthotic treatment for mechanically induced 1^st ^MTP joint pain and to investigate whether any change in pain correlated with changes in foot and ankle kinematic values, as predicted by a previously proposed mechanism of function [16]. From the efficacy analysis, the 33.5 mm difference between baseline FFI(pain) scores (48 mm) and endpoint (14.5 mm) was in excess of the 10 mm identified *a priori *for this study as clinically relevant [33]. Previous studies that have explored the level of pain reduction required on a 0-100 mm VAPS, to offer individuals an adequate analgesic response to treatment, have concluded that this requires a 30 mm reduction [27] and a 32 mm reduction [44] in pain score. The 33.5 mm reduction in pain score achieved in this study is therefore in excess of what has been advocated previously as an adequate analgesic response to treatment.

With pain reduction sustained, and even continuing to improve, over the twenty-four weeks, this study indicates that foot orthoses may have treatment effects over clinically relevant periods of time. This is in agreement with other authors who have explored the efficacy of variously produced foot orthoses for other indications [45,46]. Contrary to the hypothesised mode of action [16], the exploratory analysis revealed that the reduction in 1^st ^MTP joint pain, following orthotic intervention, was not accompanied by change in 1^st ^MTP joint dorsiflexion, nor did orthoses induce significant ankle/subtalar complex frontal plane change. It remains unclear precisely how foot orthoses may influence 1^st ^MTP joint pain.

In this study, the maximum dorsiflexion values obtained at the 1^st ^MTP joint (median = 8.34°, minimum = 1.81°, maximum = 31.07°) were lower than reported following previous investigations using similar EMT systems to measure 1^st ^MTP joint motion: 37° [22]; 42° [10]; and 38° - 40° [21]. Although our participants were selected on the basis that they possessed at least 40° non-weightbearing dorsiflexion, the lower values in our study are consistent with a cohort of participants specifically selected for 1^st ^MTP joint pain of mechanical origin and a potential for functional blockade of 1^st ^MTP joint dorsiflexion.

This study demonstrated a large kinematic variability in both the no-orthoses and the orthoses conditions. While large variation is expected with such a small sample size, this is a finding mirrored by a previous study that used intracortical pins to assess kinematic effects of foot orthoses [47], where a similar subject-specific and unsystematic effect was reported. Nester [48], from a review of recent dynamic cadaver and invasive kinematic research approaches, concluded that there was a similarly high and normal variation of foot kinematics between individuals.

The modified, prefabricated orthotic device used in this study is of a type that is being increasingly favoured over more expensive, casted devices due to evidence that there may be little functional difference between the two types of orthotic device [49].

In the absence of a control group and a randomisation protocol, we recognise that this study provides only minimal further support for the therapeutic effect of foot orthoses. It is possible that the pain reduction gained by the participants could have been related to reasons other than the therapeutic effect of the foot orthoses such as the placebo effect, a change in footwear required for the accommodation of the foot orthoses, the participant incorrectly reporting lower pain levels to please the clinician or through natural resolution of symptoms over time. We also acknowledge that the small data set for the kinematic analysis may not have been sufficient to detect the effect of orthoses on joint motion. Furthermore the results can be applied only to the specific orthotic device tested and it is not known if the results obtained would have been different for a device manufactured by a different method, and/or from different materials. Future research should employ gold standard methods and should extend the scope of the study to investigate a variety of different manufacturing and prescription methods that are commonly employed.

In the longitudinal efficacy study, participants wore their own footwear following assessment for suitability. There was however a level of variability amongst participants' footwear that may have influenced the therapeutic effect of the devices as there is a known orthotic effect of footwear alone [50,51]. For the kinematic exploratory phase, participants wore a standardised neoprene boot in the laboratory setting. We note that foot orthoses are typically worn in structured footwear though we wanted to minimise the potential confounding effect of footwear, focusing on the functional changes associated with the orthotic device alone.

Potential participants with a known traumatic aetiology or systemic disease, that could have contributed to their 1^st ^MTP joint pain, were excluded from the study. There was an assumption that those included in the study had therefore mechanically induced pain. The authors accept that there may possibly have been other unknown factors that may have contributed to the onset of symptoms.

The study focused on kinematic changes at the foot in the attempt to determine if a correlation could be drawn between changes in pain and changes in kinematics. It is acknowledged that the therapeutic effect may be due to other factors such as kinetic or temporal changes. Further research should include analysis of these variables.

Finally, the authors appreciate that the findings of this cohort study are at a hypothesis generating level and can only suggest certain trends which would inform a more robust analysis in the form of a future randomised, controlled trial. To our knowledge however, this is the first study to date that has investigated the efficacy of foot orthoses on individuals with mechanically induced 1^st ^MTP joint pain prospectively, as well as looking at associated changes in foot and ankle kinematics.

## Conclusions

The results of this study suggest that a commonly used orthotic design can offer a reduction in mechanically induced pain at the 1^st ^MTP joint to a level that is considered an adequate analgesic response to treatment. The hypothesised mode of action was not confirmed however, as pain relief was not associated with increased dorsiflexion at the 1^st ^MTP joint or reduced eversion (pronation) at the ankle/subtalar complex. Further study is required to determine definitively the efficacy of foot orthoses in the management of 1^st ^MTP joint pain and to explore the mechanism of action.

## Competing interests

The study was supported in part through an unrestricted grant from Healthy Step (Sensograph) who distribute X-line^® ^foot orthoses in the UK.

## Authors' contributions

BJW conceived the study design, undertook the clinical investigations and contributed to the data analysis and writing of the manuscript. AMK contributed to the study design, clinical and laboratory investigations and to the data analysis and writing of the manuscript. ACR contributed to the laboratory investigations and contributed to the data analysis and writing of the manuscript. NC contributed to the study design and writing of the manuscript. All authors read and approved the final manuscript.
